# Body Dissatisfaction and Its Association with Health-Related Factors in Rural and Urban Mexican Adolescents from the State of Jalisco

**DOI:** 10.3390/ijerph182212215

**Published:** 2021-11-20

**Authors:** María Rivera-Ochoa, Marta Arroyo-Bello, Asier Mañas, Carlos Quesada-González, Barbara Vizmanos-Lamotte, Marcela González-Gross, Amelia Guadalupe-Grau

**Affiliations:** 1 Department of Health and Human Performance, ImFINE Research Group, Universidad Politécnica de Madrid, 28040 Madrid, Spain; mariariveraochoa@gmail.com (M.R.-O.); carlos.quesada@upm.es (C.Q.-G.); marcela.gonzalez.gross@upm.es (M.G.-G.); 2 North University Center, Universidad de Guadalajara, Guadalajara 44430, Mexico; 3 Department of Psychology, University of Westminster, London W1B 2HW, UK; m.arroyobello@hotmail.com; 4 Department of Psychology, Universidad Nacional de Educación a Distancia (UNED), 28040 Madrid, Spain; 5 Deapartment of Physical Activity and Sports Sciences, GENUD Toledo Research Group, Universidad de Castilla-La Mancha, 45071 Toledo, Spain; asier.manas@uclm.es; 6 Carlos III Health Institute, CIBER of Frailty and Healthy Aging (CIBERFES), 28029 Madrid, Spain; 7 Department of Applied Mathematics to Information and Communication Technologies, Universidad Politécnica de Madrid, 28040 Madrid, Spain; 8 Institute of Nutrigenetics and Translational Nutrigenomics, University Center for Health Sciences, Universidad de Guadalajara, Guadalajara 44430, Mexico; bvizmanos@yahoo.com.mx

**Keywords:** body dissatisfaction, adolescents, body composition, physical fitness, physical activity, Mexico

## Abstract

Background: To better understand Mexican adolescent’s body self-perception, this study aimed to analyze their body dissatisfaction (BD) levels according to sex and place of residence. We also aimed to explore differences in body composition (BC), physical fitness (PF), and physical activity (PA) between satisfied and dissatisfied adolescents and to find the associations between BD and these parameters. Methods: Cross-sectional, observational study carried out in Jalisco (Mexico) in which 451 adolescents (43.68% males, 43.90% rural) aged 13–17 years were evaluated. BD and self-perceived PF and PA were assessed with validated questionnaires, while objectively measured PF (strength, explosive strength, speed-agility, and cardiovascular fitness) was assessed using field tests. To evaluate BC, anthropometric measures and bioelectric impedance analysis were carried out. Regression analysis was used to ascertain the associations between health-related factors and body dissatisfaction. Results: Girls obtained higher scores on the BD questionnaire than boys (11.12 ± 3.13 vs. 10.33 ± 2.73; *p* < 0.05), whereas no geographical differences between rural and urban adolescents were found. BD was positively associated with higher fat mass (β = 0.15; *p* < 0.001), and negatively associated with muscle mass and PF (β = −0.24 and β = −0.23; *p* < 0.001). However, PA was not associated with any of the measured parameters. Conclusions: selected parameters of BC, PF, and sex have an impact on the Mexican adolescent’s body satisfaction levels and should be considered when designing future health policies.

## 1. Introduction

Body dissatisfaction (BD) is defined by the negative attitudes and perceptions people have about their physical appearance. It is assumed to originate from the discrepancy between the body’s current self-perception and the ideal body shape [1]. A large part of the population is affected by this phenomenon and its consequences are a public health concern worldwide, impacting people’s psychological functioning and general wellbeing [2]. BD is a significant risk factor for developing eating disorders, overweight, obesity, and even depression [3].

Despite being psychological in nature, BD is greatly influenced by sociocultural factors [1]. The Western lifestyle is defined as a lifestyle that is high-consuming and individualistic in its nature [4] and plays an essential role in the development of BD in all cultures. Globalization and the constant exposure to Western lifestyles through social media have permeated most nonwestern societies [5]. In addition, due to its proximity to the United States, Mexico is found to be highly influenced by Western beauty standards [6] Accordingly, there is tremendous social pressure to fit into sometimes unachievable body goals [7]. This situation is particularly apparent for the girl’s portrayal of the ‘perfect body,’ which tends to be extremely thin. On the contrary, boys desire to seek larger, more muscular, and athletic bodies, as physical strength is the focus of masculinity [8,9].

Within the general population, adolescents are more vulnerable to experience BD. This fact is not surprising when looking at the multiple behavioral and physical changes puberty brings [10]. In this period, acceptance and belonging become central to the adolescent’s existence, and in consequence, seeking the ideal body and being desired by others is key to social success [6].

It must be noted that the evaluation people make of their body does not necessarily match with their actual body shape or weight [11]. However, research has found that adolescents whose body composition (BC) is worse than recommended are more prone to become dissatisfied with their bodies. This phenomenon, in turn, can result in lower self-esteem, triggers of stress, and developing risky healthy habits such as self-harm, excessive drinking, drug use, and risky diet habits [12]. Some studies have shown that despite understanding the importance of a healthy lifestyle, having BD, low self-esteem, and high stress levels can be an obstacle to changing sedentary and unhealthy habits [13].

The benefits of physical activity (PA) on adolescents’ physical and mental health have been proven extensively [14,15]. However, the relationship between BD and PA is complex. There seem to be different results according to gender and how vigorous the PA should be to create a significant difference in young people’s attitudes towards their body image [12].

In contrast, physical fitness (PF) has been positively correlated with adolescents’ body satisfaction [16]. Thus, being more robust, more flexible, and aerobically fit, or having athletic competence can contribute to feeling valued and having fewer negative perceptions of one’s body regardless of weight, body shape, or BC [17].

According to the latest Mexican National Survey on Health and Nutrition (ENSANUT) [18], the prevalence of overweight and obesity has increased in rural areas. Therefore, it would be beneficial to know if the place of residence of Mexican adolescents is linked to BD also because lifestyle differences and body size ideals might change according to where adolescents reside [19]. Moreover, sociodemographic factors in urban and rural areas manifest themselves differently and could have alternative views of oneself [20]. While some findings reveal a higher prevalence of BD in urban areas than in rural areas [21], others have found more significant concerns about weight and diet behavior within rural populations. For instance, adolescents from urban areas had higher expectations of the ideal body image than their suburban and rural counterparts. In contrast, in rural areas, more preadolescents wanted to lose weight [22]. Despite these findings in other countries, the potential role of geographical location on BD in Mexican adolescents is unknown.

Previous results obtained from our research group [23] suggested that there are differences in the BC, PA, and PF of adolescents according to their place of residence. Rural Mexican adolescents from the state of Jalisco are less sedentary than their urban peers. They present a generally higher fatness profile and a lower fitness profile of muscle power, agility-coordination and flexibility. Accordingly, these findings could also indicate a difference in BD.

Finding ways to promote healthier habits during adolescence seems crucial as the lifestyle choices acquired and the BD experienced during this period progress to adulthood. The consequences of this in society profoundly impact people’s future health and well-being [24]. Therefore, the purpose of this study was to analyze the differences in BD of Mexican adolescents according to sex and place of residence. We also aimed to explore the differences in body composition, physical fitness, and physical activity between satisfied and dissatisfied adolescents and the association between BD levels and these parameters. Our hypothesis was that rural adolescents would show lower BD levels because they are less influenced by Western societies beauty standards, and they are more physically active than their urban counterparts.

## 2. Materials and Methods

### 2.1. Study Design and Sample

The Healthy Lifestyle in Mexico by Nutrition in Adolescence (HELENA-MEX) [23] is a randomized multi-center cross-sectional study conducted in Jalisco, Mexico. Four schools of the North Region of Jalisco (rural) and four schools of the Metropolitan Area of Guadalajara (urban) participated. A final sample of 451 adolescents (43.68% boys, 43.90% rural) aged 13–17 was evaluated between January and June 2018. Adolescents and their legal guardians were previously informed about the study procedures and voluntary participation and had to sign an informed consent form. The study was approved by the Ethics, Research and Biosafety Committee of the University of Guadalajara, Mexico (CI-04717).

### 2.2. Measures

A doctor created a medical history of each adolescent to assess their health status and sociodemographic information. Data such as age, sex, and place of residence of each participant were recorded. The maturity stage was self-reported by choosing images drawn from the Tanner and Whitehouse method [25]. Each child was provided with sex-appropriate schematic drawings (breasts and pubic hair in girls; genitalia and pubic hair in boys) and requested to rate themself on each dimension. Self-ratings were averaged to yield a single individual score (ranging from I—prepubertal, to V—adult level of development).

#### 2.2.1. Body Dissatisfaction

Body image was assessed with five items of the Eating attitudes and Weight problems Inventory questionnaire [26] (EWI) of the category “Body dissatisfaction.” The chosen items of the questionnaire were the following: “I think my bottom is too fat,” “I think my stomach is too fat,” “I think my thighs are too fat,” “I think my hips are too wide” and “I am content with my figure”. The items used a four-point response format (1 = does not apply at all, 2 = seldom applies, 3 = occasionally applies and 4 = always applies). The item “I am content with my figure” was recorded (1 = 4, 2 = 3, 3 = 2; 4 = 1). The total score of Body Dissatisfaction was calculated by adding up the responses to all items. Subsequently, according to the total score obtained, adolescents were satisfied or dissatisfied with their body image. With a five-point score being the lowest possible and 20 the highest, the cut-off point was 12.5. Participants with a score of 12.5 or lower were classed as satisfied and the remaining as dissatisfied [27].

#### 2.2.2. Body Composition

To assess BC, anthropometric measures and bioelectric impedance analyses were carried out. The measurements were taken after overnight fasting, with light sports clothing and without shoes. A stadiometer was used to measure participants’ height to the nearest 0.1 cm (SECA 215, Hamburg, Germany). A precision digital scale was used to measure body weight or mass to the nearest 0.1 Kg and to perform the bioelectric impedance analysis (Inbody 120, BioSpace Co., Seoul, Korea). The Body Mass Index (BMI) was calculated as weight/height^2^ (kg/m^2^). Percent fat mass (FM) and percent skeletal muscle mass (SMM) were calculated from the Inbody120 software as described before [23].

#### 2.2.3. Physical Activity

A questionnaire about PA (IPAQ), previously validated in the adolescent population [28], was administered to assess the levels of PA. Participants were asked about their PA in the previous seven days and the PA duration (hours and minutes per day), frequency (times per week), and intensity (light, moderate, and vigorous) was scored according to the original guidelines (The IPAQ Group). MVPA was analyzed because of its robust association with physical, social, and mental health outcomes in adolescents [29,30].

#### 2.2.4. Physical Fitness

PF was evaluated through four tests described in the HELENA study [31] to analyze the relationship between BD and the different physical abilities. The handgrip test assessed muscular fitness in the upper body. The Counter Movement Jump, CMJ, determined the explosive strength. The speed-agility was evaluated with the 4 × 10 m shuttle run test, SRT. A 20 m shuttle run test evaluated the aerobic fitness, and the VO2max was estimated by the equation from Leger et al. [32]. Before these tests, participants completed five questions about their self-perceived PF through the International Fitness Scale questionnaire (IFIS) [33]. Only one item was used for the current study: your general physical fitness is (1 = very poor, 2 = poor, 3 = fair, 4 =good, 5 = very good).

### 2.3. Statistical Analyses

To analyze if there were differences between rural and urban adolescents as well as between girls and boys, a Mann–Whitney U test was performed. Chi-square tests were carried out to analyze the prevalence of boys and girls in each item in which the BD was evaluated. Since the assumption of normality did not hold for most variables, Mann–Whitney U tests were also conducted to evaluate the differences between BC, PF, and PA according to the adolescents’ body image satisfaction or dissatisfaction.

Linear regressions were carried out to analyze how the studied variables were associated with BD in adolescents. One linear regression was carried out for each of these variables as the independent variable and setting BD as the dependent one and it was conducted again adjusting sex, age, and maturity stage. The relation was evaluated using R2, and Cohen’s f2 was used to study the effect size, following the criteria in [34]; 0.02—small effect, 0.15—medium, and 0.35—large. Multiple linear regression analyses were also considered, although no relevant results were found, and thus, they were not reported.

The significance level was set at 0.05 for all tests, and all statistical analyses were performed using SPSS v.25.0 (SPAA Inc., Chicago, IL, USA).

## 3. Results

Descriptive statistics for BD by sex are reported in Table 1. No significant differences were found in the item: “I think my hips are too wide” and a significantly higher percentage of girls than boys answered that they thought their bottom, stomach, and thighs were too fat (*p* < 0.05). A total of 46.9% of the girls indicated that they were not content with their figure compared to 31.5% of the boys (*p* < 0.05). Girls had a significantly higher BD score than boys (mean 11.12 ± 3.13 vs. 10.33 ± 2.73 respectively; *p* < 0.05). According to residence, only girls from the urban population presented higher BD than their rural counterparts (Z = −2.52; *p* < 0.05).

### 3.1. Differences between Body Dissatisfaction Groups

Body composition, physical performance, and physical activity levels according to sex and body self-perception are depicted in Table 2. Adolescents classified as dissatisfied with their bodies presented a significantly higher BMI and FM and a lower SMM than the participants who were classified as satisfied (*p* < 0.001). When the self-perceived PF of adolescents was better, they had more body satisfaction (*p* < 0.001). Likewise, when levels of general PF (*p* < 0.001) and explosive strength (*p* < 0.001) were higher, their body satisfaction was also better. There were no significant differences in the speed-agility and aerobic fitness tests when the data were divided by sex. However, there was a significant difference in the total sample. Adolescents with more speed-agility and higher aerobic fitness showed higher levels of body satisfaction (*p* < 0.05). We did not find significant differences in upper-body strength or MVPA weekly time between both groups (*p* = 0.559 and *p* = 0.508, respectively) (Table 2).

### 3.2. Association between BD and Body Composition

There was no interaction in terms of sex in the linear regression analyses; the results are, therefore, presented as the total sample. The significant indexes, statistical power, and size effect of the linear regression tests performed are shown in Table 3. A large association between BC and BD was found. Higher BMI and FM percentages were associated with higher levels of BD (β = 0.36 and β = 0.15, respectively; *p* < 0.001) and higher SMM percentages were associated with lower levels of BD (β = −0.24; *p* < 0.001).

### 3.3. Association between BD, Physical Function, and Physical Activity

Table 3 shows that better results in self-perceived PF, general PF, explosive strength, speed-agility, and aerobic fitness were associated with lower levels of BD, showing a moderate size effect (β = −1.19, β = −0.23, β = −0.14, β = 0.38 and β = −0.09, respectively; *p* < 0.001). No association was observed for upper-body muscular strength and MVPA (β = −0.01, *p* = 0.483 and β = −0.00; *p* = 0.082) (Table 3). The size effect improved in all associations when adjusting the analyses by sex, age, and maturity stage (Tanner).

## 4. Discussion

The aim of this study was two-fold. Firstly, to learn more about BD among boys and girls within rural and urban areas in Mexico, and secondly, to explore different factors that can impact this phenomenon. Girls had a higher score in BD than boys, while no differences between rural and urban adolescents were found. Higher BMI and FM percentages were associated with higher levels of BD, and higher SMM percentages were associated with lower levels of BD. Better results in self-perceived PF, general PF, explosive strength, speed-agility, and aerobic fitness were associated with lower levels of BD. No association was observed for MVPA.

### 4.1. Place of Residence

Regarding the place of residence, no differences were found between the BD of adolescents who live in rural areas and those in urban areas. Even though there is little research that analyzes BD in connection with differences in culture, ethnicity, and educational and socio-economical levels [35], our results are supported by research carried out in other countries such as Brazil [21,36,37]. Nevertheless, when Western lifestyle influences adolescents, they seem to be exposed to the same information regarding body image. These messages can come from family, friends, and the media independently of cultural and socioeconomic differences [38].

### 4.2. Sex and Body Composition

This study has found higher levels of BD in girls than in boys. Accordingly, in 2017, Jimenez-Florez [35] published a systematic review that concluded that BD has a higher impact in girls than in boys. Most findings emphasize BC as a crucial variable when determining BD in adolescents [39]. It is known that the ideal body is presented differently according to sex [40]. Boys who are dissatisfied with their bodies are more inclined to increase their body size, whereas girls tend to seek the opposite effect [9,41]. In support of this, the study found that girls who were satisfied with their bodies had a lower BMI than boys in that category. In contrast, boys were more satisfied with their bodies when their muscular mass percentage was higher and their body fat percentage was lower, that is, when they had more athletic and defined bodies. Consequently, this may be the reason why female adolescents tend to do more PA aimed at losing weight while boys look for activities that focus on physical strength [8].

### 4.3. Physical Activity

Despite the importance of PA in adolescents’ health, no differences were found in the MVPA time between satisfied and dissatisfied adolescents. Likewise, it was not possible to associate BD with PA in our study. Different studies consider BD to be a barrier for the practice of MVPA [42,43,44], so it could be expected that higher levels of BD would mean less time for MVPA. However, there is also evidence from previous research that shows how regular and vigorous PA improves body satisfaction [39,45]. BD can motivate the practice of MVPA in adolescents who want to reduce their shape, and at the same time, it has been found that BD combined with weight criticisms are linked with lower levels of PA in adolescents [46].

### 4.4. Physical Fitness

Another factor related to adolescents’ health is PF. Our results have shown that BD is associated with self-perceived PF and objective measured PF. In line with these findings, the more satisfied adolescents feel about their PF, the more content they are with their body image [47,48]. Nevertheless, this is influenced by the ideal body image set by society. The thin and lean body standards for girls [7] may not be linked to being more agile or having a healthier cardiorespiratory system. For instance, adolescents who wanted a thinner body presented more BD when their levels of cardiorespiratory fitness were lower. In contrast, adolescents who wanted to increase their body size showed more BD when they had lower levels of upper-body strength [49]. On the one hand, PF is greatly influenced by the ideal BC of each adolescent. However, on the other hand, perception of adolescents’ fitness and exercise self-efficacy contributes to physical confidence, physical fitness abilities, and sports competence [47], boosting their BD.

### 4.5. Strengths and Limitations

There is little scientific data about the factors that impact BD in Mexican adolescents. Our study sets a precedent for future research. A large number of studies evaluate the relationship of BD with PA and PF through self-report. However, our study contributes to the evaluation of PF objectively and evaluates PA using a validated questionnaire. The current research has a number of limitations. Firstly, there is a large variety of methodologies to evaluate BD. Although the one used for our study is validated, it is impossible to elaborate clear conclusions when comparing our results with others found in the literature. It is needed to standardize the evaluation criteria of BD in order to make comparisons between different populations and studies. Secondly, self-administered questionnaires were used to report PA. However, the use of accelerometers would have been preferable to obtain objective measures and to help clarify the relationship between PA and BD. Thirdly, the evaluation of BD done in our study does not allow us to differentiate between adolescents who desire a thinner silhouette and who prefer a larger body. Taking this into account would help explain the relationship of BD with PF and PA and obtain more detailed information about its relationship with BC. Finally, future research would benefit from these results by considering the limitations and strengths stated to expand on the research about the factors involved in BD.

## 5. Conclusions and Practical Applications

The present findings determined that selected BC and PF variables influence the body satisfaction of Mexican adolescents, regardless of their geographical location. Evidence from the present investigation provides some potential points of guidance for interventions and public health policies aimed at tackling the increasing obesogenic environment of Mexican adolescents. First, obesity-related public health media campaigns should focus on behavioral change and not on weight per se, as well as on the potential health benefit that could be achieved when weight loss is achieved incorporating physical exercise to the interventions. Second, body image should be specifically addressed and evaluated to ensure that there have been no adverse effects and to assess for positive impact. Third, funding sources should require that investigators clearly address the role of body image and potential adverse consequences, as well as how these factors are to be assessed.

## Figures and Tables

**Table 1:** Differences between sex and place of residence in body dissatisfaction.

	Total (*n* = 451)	Boys (*n* = 197)	Girls (*n* = 254)
**Answer sometimes or always true**			
I think my bottom is too fat	14.9%	13.2%	16.1% *
I think my stomach is too fat	41.9%	33.5%	48.4% *
I think my thighs are too fat	32.6%	31.5%	33.5% *
I think my hips are too wide	27.1%	23.4%	29.9%
I am content with my figure ^1^	40.1%	31.5%	46.9% *
**Score body dissatisfaction**	10.77 ± 2.29	10.33 ± 2.73	11.12 ± 3.13 ^§^
Rural (*n* = 198)	10.60 ± 2.91	10.21 ± 2.81	10.83 ± 2.96
Urban (*n* = 253)	10.91 ± 3.04	10.40 ± 2.70	11.40 ± 3.27 ^§^

Note: ^1^ This item was recoded. Higher prevalence would mean higher body dissatisfaction. * Chi-square tests: *p* < 0.05. ^§^ Mann–Whitney U test: *p* < 0.05.

**Table 2:** Differences between body dissatisfaction groups of adolescents in selected variables.

	Total				Boys				Girls			
	Dissatisfaction		Satisfaction		Dissatisfaction		Satisfaction		Dissatisfaction		Satisfaction	
	*n*	Mean ± SD	*n*	Mean ± SD	*n*	Mean ± SD	*n*	Mean ± SD	*n*	Mean ± SD	*n*	Mean ± SD
**Body Composition**												
BMI	115	25.36 ± 4.88	329	20.97 ± 3.59 **	42	24.99 ± 5.57	150	21.08 ± 3.78 **	73	25.57 ± 4.47	179	20.88 ± 3.42 **
Fat Mass (%)	114	32.88 ± 9.24	327	24.43 ± 9.56 **	42	26.18 ± 8.18	148	18.12 ± 7.93 **	72	36.79 ± 7.42	179	29.64 ± 7.41 **
SMM (%)	114	36.77 ± 5.12	327	41.25 ± 5.62 **	42	40.93 ± 4.19	148	45.42 ± 4.42 **	72	34.34 ± 3.91	179	37.8 ± 3.9 **
**Physical Fitness**												
Self-perceived PF	116	2.89 ± 0.91	334	3.5 ± 0.88 **	43	3.09 ± 0.97	153	3.7 ± 0.89 **	73	2.77 ± 0.86	181	3.34 ± 0.84 **
PF	93	-1.02 ± 2.63	280	0.32 ± 3.19 **	33	1.44 ± 2.3	129	2.85 ± 2.6 *	60	−2.38 ± 1.63	151	−1.84 ± 1.7 *
Handgrip (kg) ^1^	93	35.25 ± 6.26	286	37.41 ± 6.7	33	39.12 ± 6.99	131	41.22 ± 6.98	60	33.12 ± 4.65	155	34.19 ± 4.39
CMJ (cm)	109	19.2 ± 5.13	312	22.33 ± 6.19 **	40	22.52 ± 5.11	148	25.68 ± 6.27 *	69	17.28 ± 4.08	164	19.31 ± 4.28 *
4 × 10 m SRT (s)	109	13.46 ± 1.63	310	12.88 ± 1.57 **	40	12.34 ± 1.57	147	11.82 ± 1.14	69	14.11 ± 1.27	163	13.84 ± 1.27
VO2max (mL/kg/min)	108	27.3 ± 7.23	312	27.71 ± 8.07 *	40	31.79 ± 8.48	148	32.6 ± 7.96	68	24.66 ± 4.74	164	23.29 ± 5.06
**Physical Activity**												
MVPA (min/wk)	77	510.01 ± 457.55	250	568.34 ± 470.71	27	484.78 ± 439.94	112	656.26 ± 490.64	50	523.64 ± 470.6	138	496.99 ± 442.96

Note: BMI = Body Mass Index; SMM = Skeletal Muscle Mass; PF = Physical Fitness; CMJ = Counter Movement Jump; SRT = Shuttle Run Test; VO2max = maximal oxygen consumption; MVPA = Moderate to Vigorous Physical Activity. ^1^ Handgrip is expressed as mean of right and left side. * Mann–Whitney U test: *p* < 0.05. ** Mann–Whitney U test: *p* < 0.001.

**Table 3:** Lineal regressions between the body dissatisfaction (BD) score of adolescents in selected variables.

	Models				Models Adjusted ^&^			
	*n*	β (IC95%)	R^2^	f^2^	*n*	β (IC95%)	R^2^	f^2^
**Body Composition**								
BMI	444	0.36 (0.30, 0.41) **	0.28	0.38	442	0.36 (0.31, 0.42) **	0.30	0.43
Fat Mass (%)	441	0.15 (0.12, 0.17) **	0.24	0.32	439	0.19 (0.16, 0.21) **	0.28	0.38
SMM (%)	441	−0.24 (−0.28, −0.19) **	0.21	0.27	439	−0.33 (−0.39, −0,27) **	0.26	0.36
**Physical Fitness**								
Self-perceived PF	450	−1.19 (−1.47, −0.92) **	0.14	0.16	448	−1.16 (−1.45, −0.87) **	0.15	0.18
PF	373	−0.23 (−0.33, −0.13) **	0.06	0.06	372	−0.27 (−0.41, −0.13) **	0.07	0.08
Handgrip (kg) ^1^	420	−0.01 (−0.05, 0.02)	0.00	0.00	418	0.02 (−0.02, 0.07) *	0.04	0.04
CMJ (cm)	421	−0.14 (−0.18, −0.09) **	0.08	0.08	419	−0.14 (−0.19, −0.09) **	0.10	0.10
4 × 10 m SRT (s)	419	0.38 (0.21, 0.56) **	0.04	0.04	417	0.35 (0.13, 0.58) **	0.06	0.06
VO2max (mL/kg/min)	379	−0.09 (−0.14, −0.05) **	0.04	0.04	378	−0.08 (−1.13, −0.03) **	0.06	0.06
**Physical Activity**								
MVPA (min/wk)	327	−0.00 (0.00, 0.00)	0.01	0.01	325	0.00 (−0.00, 0.00) *	0.05	0.05

Note: BMI = Body Mass Index; SMM = Skeletal Muscle; PF = Physical Fitness; CMJ = Counter Movement Jump; SRT = Shuttle Run Test; VO2max = maximal oxygen consumption; MVPA = Moderate to Vigorous Physical Activity. ^1^ Handgrip is expressed as mean of right and left side. * linear regression: *p* < 0.05. ** linear regression: *p* < 0.001. f^2^ effect size: 0.02—small, 0.15—medium, 0.35—large. ^&^ Adjusted by sex, age, and maturity stage.
